# Orthodontic treatment of periodontal patients: challenges and solutions, from planning to retention

**DOI:** 10.1590/2177-6709.25.6.079-116.sar

**Published:** 2020

**Authors:** Daniela Feu

**Affiliations:** 1 Universidade Vila Velha, Departamento de Odontologia, Disciplina de Ortodontia (Vila Velha/ES, Brazil).

**Keywords:** Adult periodontitis, Orthodontics, Anchorage

## Abstract

**Introduction:**

There is an increasing number of adult patients with sequelae of periodontal diseases seeking orthodontic treatment to improve their occlusion and quality of life. However, it is important to highlight that the patient who has vertical bone loss has unique needs, arising from the frequent related pathological migrations. Therefore, it requires an individualized orthodontic treatment in terms of anchorage, biomechanics, and multidisciplinary planning, which raises doubts in the hierarchy of priorities and organization of the treatment plan.

**Objectives:**

It was proposed a stratified hierarchy of the needs of orthodontic-periodontal treatment in six levels, which were illustrated with examples of clinical cases in which biomechanical planning and a multidisciplinary approach made it possible to obtain a balanced occlusion, aesthetic improvement and stabilization of the results.

**Conclusion:**

Orthodontic treatment of periodontal patients with a multidisciplinary approach is increasingly frequent and should be planned individually, considering bone losses suffered by each patient. Respecting some limitations, it is possible to improve the level of bone insertion, smile aesthetics and masticatory function, to facilitate oral hygiene through the orthodontic treatment of adult patients with little bone support. It is also important to highlight that there are unique aspects in the orthodontic retention in these cases.

## RESUMO

**Introdução:**

É crescente o número de pacientes adultos portadores de sequelas de doenças periodontais que buscam tratamento ortodôntico para melhorar sua oclusão e qualidade de vida. Contudo, é importante destacar que o paciente que apresenta perda óssea vertical possui necessidades únicas, advindas das frequentes migrações patológicas relacionadas. Logo, requer um tratamento ortodôntico individualizado quanto a quesitos de ancoragem, biomecânica e planejamento multidisciplinar, o que gera dúvidas na hierarquização de prioridades e organização do plano de tratamento.

**Objetivos:**

Foi proposta uma hierarquização estratificada das necessidades do tratamento ortodôntico-periodontal em seis níveis, os quais foram ilustrados com exemplos de casos clínicos nos quais o planejamento biomecânico e a abordagem multidisciplinar possibilitaram a obtenção de uma oclusão equilibrada, melhora estética e estabilização dos resultados obtidos.

**Conclusão:**

O tratamento ortodôntico de pacientes periodontais com abordagem multidisciplinar é cada vez mais frequente e deve ser planejado de forma individualizada, considerando as perdas de inserção sofridas por cada paciente. Respeitando-se algumas limitações, é possível melhorar o nível de inserção óssea, a estética do sorriso e a função mastigatória, além de facilitar a higiene bucal, por meio do tratamento ortodôntico de pacientes adultos com pouco suporte ósseo. Também é importante destacar que há aspectos únicos na contenção ortodôntica desses casos.

## INTRODUCTION

With an increasing number of adult patients seeking orthodontic treatment, it is now common to treat patients who need correction of functional and esthetic sequelae of periodontal disease.^1^ In fact, periodontal treatment as a single therapy is not always able to correct and control the damage produced by periodontal disease and a consequent pathological occlusion, depending on the degree of tissue impairment. In these cases, orthodontic tooth movement is an important step when planning the global treatment of the patient,^2^ since one of the most important factors for periodontal balance is the physiological stimulation received by the tissues during function.^3^ ^,^ ^4^

Thus, recovery of the integrity and continuity of the dental arch, as well as balanced tooth positioning, is an essential step for the successful treatment of a patient with periodontitis and pathological migration of teeth.^4^ The first step in treating these patients is always the elimination of active periodontal disease.^1^ ^,^ ^3^ ^-^ ^15^ However, even after complete removal of the disease, the patient with pathological migration should not be considered completely treated.

Patients with periodontitis often have several sequelae, such as: 1) inadequate angulation; 2) excessive buccal projection; 3) extrusion of one or more incisors; and 4) development of single or multiple diastemas in anterior teeth, with progressive spacing of incisors, often fan-shaped.^1^ ^,^ ^3^ ^,^ ^4^ ^,^ ^14^ This spacing is the most evident sign of pathological change in tooth positioning, occurs in a progressive manner and the most affected teeth are the maxillary incisors.^3^ ^,^ ^4^ Concomitantly, periodontal patients have traumatic occlusion, which can contribute to the development of destructive periodontal disease, since secondary occlusal trauma may further deteriorate the supporting tissues.^4^ ^,^ ^5^

This occurs because it is extremely important that masticatory forces are transmitted in the axial direction of teeth, allowing the application of intense forces with less tension on the periodontal ligament.

When there are pathological changes in tooth positioning,^1^ ^,^ ^3^ ^,^ ^4^ ^,^ ^13^ masticatory forces start to occur in inclined planes, which causes dental hypermobility and thickening of the periodontal ligament. This is a secondary occlusal trauma caused by the reduced capacity of the periodontium to withstand normal occlusal loads,^5^ ^,^ ^16^ which contributes to the progression of pathological migration of teeth, even in the absence of active periodontal disease.^2^ ^,^ ^4^ ^,^ ^5^ This type of trauma does not cause periodontal pocket or gingivitis, nor does it increase the gingival fluid.^16^ However, it is essential to eliminate it to extinguish the inflammatory process and allow spontaneous regeneration of the periodontal ligament.^17^ In summary, secondary occlusal trauma is an aggravating factor for periodontal problems and should be eliminated by orthodontic correction.^11^

In cases of pathological migration and extrusion, the intrusion movement is recommended to realign the teeth, improve the clinical crown length and marginal bone levels.^8^ ^,^ ^14^ ^,^ ^18^ A study that analyzed histological sections of animal tissues suggests that orthodontic intrusion may allow the formation of new healthy periodontal insertion tissue.^8^ In fact, there is evidence that the combination of orthodontic intrusion with periodontal therapy has a noticeable effect on incisors that suffered pathological migration caused by periodontitis.^1^ ^,^ ^10^ ^,^ ^13^ ^,^ ^18^ This combination can achieve several benefits: stabilization or recovery of alveolar bone height; correction of dental positions; stabilization of new positions by splints; and significant improvement in facial profiles.^1^ ^,^ ^14^ ^,^ ^18^

## TREATMENT PLANNING - THE PYRAMID OF ORTHODONTIC-PERIODONTAL PLANNING

In these interdisciplinary cases, orthodontic therapy should be focused on eliminating or reducing the severity of periodontitis sequelae. However, planning of orthodontic-periodontal treatment usually raises doubts among orthodontists, since it involves a conflict of priorities between the specialties involved. To create a way to stratify and prioritize the planning and treatment needs of a patient with vertical bone loss, the “Pyramid of Esthetics Needs of Smile”^19^ was used as reference, which stratifies the esthetic needs in the search for an ideal smile into four levels.

**Figure 1:**
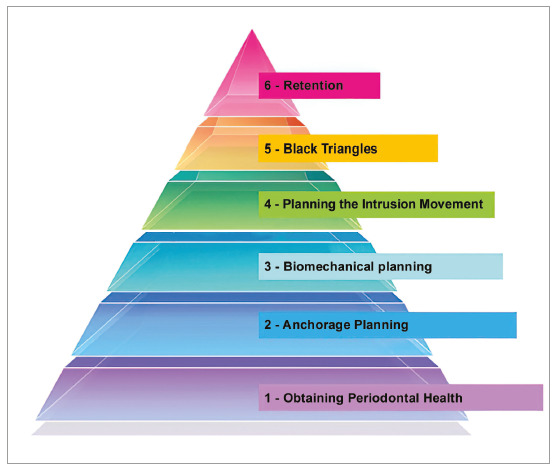
Pyramid of Orthodontic-Periodontal Planning.

Similarly, a Pyramid of Orthodontic-Periodontal Planning was designed (Fig. 1), stratified into six stages of the sequence of individualized planning of the patient with periodontitis. The ascending order of levels in the pyramid does not determine its importance;^19^ it is based on the sequence used by most orthodontic-periodontal studies.^2^ ^-^ ^4^ ^,^ ^6^ ^-^ ^9^ ^,^ ^10^ ^-^ ^12^ ^,^ ^14^ ^,^ ^18^ ^,^ ^20^ ^-^ ^23^

## THE SIX STAGES IN THE SEQUENCE OF ORTHODONTIC-PERIODONTAL PLANNING

1) The base of the pyramid - Level 1: obtaining periodontal health. The accumulation of dental biofilm is the most important factor in the initiation, progression and recurrence of periodontal disease.^5^ Longitudinal studies by Re et al.^6^ (12 years) and Boyd et al.^7^ demonstrated that orthodontic therapy is indicated for the treatment of occlusal sequelae in patients with controlled severe periodontitis. After controlling the periodontal disease, teeth with insertion loss may be submitted to movement. Orthodontic treatment can even improve bone support and the prognosis of stabilizing periodontal results in the long term.^1^ ^,^ ^2^ ^,^ ^4^ ^,^ ^6^ However, the periodontium only responds favorably to orthodontic movement if it is healthy. Therefore, the first step in treating a patient with bone loss is to check whether periodontitis has been properly controlled by a periodontist.^3^ ^-^ ^15^ ^,^ ^20^ Orthodontic movement can only be initiated after obtaining control of inflammation and dental biofilm, with excellent cooperation from the patient regarding oral hygiene techniques. This stage can last from 3 to 9 months, depending on the severity of the problem.^6^ ^,^ ^7^ ^,^ ^11^

Intensive periodontal treatment involves instructions for oral hygiene, removal of biofilm retentive factors, and scaling and root planing, which may include flap surgery and/or antibiotic prescription.^13^ ^,^ ^18^ Before the onset of orthodontic treatment, periodontal disease must be controlled. In principle, in the initial treatment stage, only teeth with very severe conditions that preclude the control of inflammation^10^ ^,^ ^12^ ^,^ ^13^ ^,^ ^20^ should be extracted. If a tooth has a prognosis of extraction due to marked insertion loss, yet it presents inflammation under control, it can be maintained during orthodontic treatment, since it facilitates anchorage and provides greater comfort to the patient.^10^ ^,^ ^11^ ^,^ ^20^

In addition to the periodontal disease control, it is also essential to maintain the results obtained by periodic control by the periodontist, at intervals of 1 to 4 months. This time interval depends on the quality of biofilm control obtained by the patient. These periodontal maintenance consultations are essential for the periodontist to evaluate and control sites that may have recurrence of the inflammatory process, to maintain periodontal health^7^ ^-^ ^9^ ^,^ ^11^ ^,^ ^21^ and also to reinforce the hygiene instructions.

The appliances used in the orthodontic treatment of these patients should not favor the accumulation of dental biofilm. The use of metal ligatures or self-ligating brackets and careful removal of excess resin around the brackets are recommended.^22^

In the initial planning, in this first stage, all biomechanical and anchorage needs inherent to the case are not always identified. It is common to observe that, after reestablishment of periodontal health, the problems at the pyramid base are eliminated, and the demands of higher levels become more important.

2) Level 2 - anchorage planning: The planning of movement anchorage is critical in patients with controlled periodontitis. It is difficult to obtain adequate anchorage for orthodontic movement in patients with partial edentulism and reduced alveolar bone support.^23^ As in most cases, there is a need for intrusion and retraction of the extruded and projected incisors, with diastemas and changes in angulation.^1^ ^,^ ^3^ ^,^ ^4^ ^,^ ^15^ It is mandatory to analyze if the posterior teeth of the patient have enough periodontal insertion to allow them to anchor the movement. It should also be considered that there is an increase in the extrusive component of posterior teeth with bone loss, which represents an additional component that must be considered in anchorage planning.^8^ ^,^ ^22^ ^-^ ^25^ As previously described, the anchorage must be reevaluated after achieving periodontal health, so that the condition of posterior teeth is accurately diagnosed.

When the posterior teeth are healthy and did not suffer significant insertion losses, they can be used for anchorage control in vertical and anteroposterior directions,^8^ ^,^ ^24^ ^,^ ^25^ by the use of high-pull headgear^8^ or other devices that reduce extrusion and mesial displacement of posterior teeth, such as the rigid transpalatal bar,^8^ ^,^ ^24^ the Nance button^25^ or a Bite Block^25^. However, if several posterior teeth are absent or have an unfavorable prognosis, it should be reassessed whether there is indication of extraction of the affected teeth after periodontal health is achieved.

In this case, in the absence of posterior teeth, before starting orthodontic treatment, dental implants with provisional crowns must be placed to provide anchorage. If it is not possible to use the posterior teeth as anchorage units, but there is also no indication for their extraction, absolute anchorage can be used with the aid of temporary anchoring devices (TADs).^24^ The use of absolute anchorage with TADs is highly applicable in these cases, since it provides greater safety for movement and has become a clinical reality.^21^ ^,^ ^23^ Using molars with insertion loss greater than 4mm as anchorage units may compromise their viability in the oral cavity.^23^

It is important to highlight that, in cases of severe bone loss in the anterior or posterior segments, the use of intermaxillary elastics with Class II or III direction promotes greater extrusion of these teeth, which can harm the occlusion, besides having a deleterious effect in the increase of vertical dimension.^24^ Therefore, the use of elastics should be carefully considered; it is recommended to use extra-alveolar mini-implants for sagittal corrections whenever possible. In cases in which elastics are essential, the entire posterior extent of arches must be used to reduce the extrusive component and follow the patient in intervals shorter than four weeks.^11^

3) Level 3 - biomechanical planning: The greatest limitation in the treatment of patients with controlled periodontitis is the reduction in vertical height of the alveolar bone, which alters the biomechanics of movement.^26^ ^,^ ^27^ With bone loss, the crown-root relationship is altered: the lower the bone height, the more the fulcrum of movement moves to the root apex.^7^ ^,^ ^15^ ^,^ ^18^ ^,^ ^26^ Therefore, many problems in planning the force system to be applied must be analyzed. Some important points are the following:

a) The force magnitude must be reduced proportionally to the amount of alveolar bone height of teeth to be moved.^15^ ^,^ ^18^ In the initial stage of orthodontic treatment for adults with no loss or minimal vertical bone loss, a force of 20-30g per tooth is recommended. Thereafter, the force can be increased to 30-50g (tilt movement) and 50-80g (body movement), depending on the degree of marginal bone loss and the quality of remaining alveolar bone.^28^ According to Melsen et al.,^8^ the ideal intrusion force in teeth with bone loss is between 5 and 10g per tooth.

b) With the change in positioning of the Center of Resistance (CRes), which becomes more apical as the alveolar bone height is reduced, there is a change in the moment/force ratio (M/F).^15^ ^,^ ^26^ Therefore, it is suggested to bond the orthodontic accessories as cervically as possible, provided it does not interfere with hygiene procedures. In addition, the most frequent occurrence of uncontrolled inclination movements and the greatest difficulty in achieving body movements should be highlighted.^15^ ^,^ ^26^

It is agreed that the mean perpendicular distance from the CRes to the line of action of forces that will be applied to the brackets is approximately 10 mm^29^ ^,^ ^30^ in teeth with a healthy periodontium. The application of force to the bracket 10 mm distant from the CRes of a tooth causes a moment resulting from the force and the tooth tilts, rotating around its center of rotation (CRot), whose location is usually slightly above the CRes, being altered depending on the relationship between the force action line and the CRes.^31^ Therefore, when there is alveolar bone loss, the CRes is apically displaced proportionally to the amount of bone height present, thus becoming proportionally more distant than the conventional 10 mm in relation to the bracket. Therefore, the moment (the rotation that occurred) will be greater.^15^ ^,^ ^26^

This type of movement, in which the crown moves in the direction of force and the apex moves in opposite direction to a lesser extent, is called uncontrolled inclination.^31^ Clinically, this movement occurs in patients with intact periodontium, during the retraction stage in the continuous archwire mechanics, when there is a gap at the wire/bracket interface.^31^ With the apical displacement of the CRes, this inclination becomes frequent even with the force application by thicker archwires. For this reason, the use of continuous archwires to perform intrusion and retraction movements and the use of thin, round archwires for alignment and leveling should be avoided in the treatment of periodontal patients. To obtain body movements, in these cases, the use of lever arms and segmented mechanics directed to the CRes of teeth to be moved is an important resource.^24^

The movement of continuous alignment and leveling with flexible round archwires will produce a greater tendency of projection of incisors with loss of alveolar bone height, which can be increased in the presence of a marked Spee curve. In these cases, the “relative” leveling achieved by the extrusion of posterior teeth and projection of anterior teeth^32^ will result in a greater uncontrolled inclination in the anterior region and greater posterior extrusion,^24^ depending on the magnitude of bone loss that occurred in that region, which leads to an uncontrolled force system and undesirable effects. For these reasons, it should be avoided.

c) There is a greater risk of tissue damage if a greater amount of displacement is performed.^27^ With the apical displacement of the CRes, the uncontrolled inclination becomes more frequent and generates higher pressures in the periodontium than the controlled inclination.^27^ In addition, the force will be dissipated in a smaller area of periodontium. This combination will greatly alter the dental angulations and generate unnecessary movements of the root apices, concentrating a lot of pressure in the apical region.^18^ ^,^ ^33^

With this overload of force in the apical region, the risk of resorption increases.^11^ ^,^ ^14^ ^,^ ^34^ This information must be considered together with the root anatomy and trauma history^30^ for planning more extensive movements, in which the risk of root resorption is greater. Shen et al.,^14^ in studies on patients with bone loss due to periodontal reasons, demonstrated that there is higher rate of external root resorption in lower and upper incisors, due to intrusion and retraction movement, usually performed on these teeth for correction of pathological migrations. The authors observed that the alveolar bone loss further concentrated the forces in the apical region of these teeth, which was confirmed by the finite tooth study by Choi et al.^15^ Therefore, it is essential to use light forces and adequate biomechanics in the treatment of patients with controlled periodontitis.

Similarly, the treatment must be as short and simple as possible: it is necessary to evaluate which orthodontic movements will benefit the periodontium, if conventional orthodontic objectives are applicable to each case or if there is an individual physiological limit.

d) The extrusion movement has been advocated as an effective method for: handling of one- and two-wall infrabony defects; reducing the probing depth of periodontal pockets; increasing the area of attached adhered gingiva; bone development for implant placement; and the position of the gingival margin, being considered a beneficial movement in the planning of periodontal repair.^35^ ^,^ ^36^

4) Level 4 - planning the intrusion movement

The orthodontic intrusion of teeth extruded by pathological migration is frequent in patients with vertical bone loss.^14^ Some biomechanical protocols can be proposed for intrusion and retraction of over-projected incisors due to severe loss of periodontal insertion. Ideally, the retraction forces should be directed, combined with an intrusive component, as close as possible to the center of resistance of the teeth affected by the loss of periodontal insertion. Thus, the movement becomes more effective and the results will be obtained more quickly, with low force intensity. A minimum force intensity, compatible with the intrusion movement in teeth with compromised insertion, should be used, with a maximum of 10 g per tooth.^8^ It is essential to measure the force at each orthodontic maintenance consultation, which must be performed at intervals of six weeks, to allow for a longer period of tissue repair.^8^

To achieve intrusion in teeth with a marked tendency to uncontrolled inclination (due to loss of insertion), the force must be directed to the CRes of the tooth^8^ ^,^ ^9^ ^,^ ^10^ ^,^ ^13^ ^,^ ^15^ ^,^ ^18^ ^,^ ^21^ or group of teeth that should be moved. Thus, in the case of intrusion of anterior lower dental blocks, the CRes of the movement unit should be located^29^ and its apical alteration should be estimated according to the amount of bone loss.^15^ In case of individual tooth movements, this must be done for each tooth.^15^ ^,^ ^24^ ^,^ ^29^ ^,^ ^31^

The orthodontic intrusion can displace the supragingival plaque to the subgingival region and result in the formation of infrabony periodontal pockets.^5^ However, in the absence of biofilm due to excellent oral hygiene, orthodontic intrusion associated with adequate periodontal treatment has improved the health of the reduced periodontium,^20^ ^,^ ^21^ increasing the level of periodontal insertion,^18^ ^,^ ^37^ especially in anterior teeth.^18^ Therefore, the possibility of strict scaling and root planing in the patient should be analyzed with the periodontist, to eliminate the presence of dental biofilm and granulation inflammatory tissue, ten days^7^ before the onset of active orthodontic movement. There is evidence that this procedure (which may be surgical or not) can improve the likelihood of increased periodontal insertion. When an exposed area of the root cementum is moved towards the bone, local proliferation of cells of the periodontal ligament can occur.^7^ ^-^ ^9^ ^,^ ^21^

If this procedure is not indicated, strict control of the patient's hygiene should be maintained during the intrusion movement, with monthly consultations for periodontal control.

5) Level 5 - black triangles and gingival recessions

Periodontitis can cause loss of interdental papillae, also called “black triangles” or black spaces.^38^ Besides resulting in images without esthetic harmony^19^ and causing phonetic changes, such losses contribute to the retention of food debris, affecting the health of periodontal tissues.^38^ Very often, after the closure of diastemas resulting from the pathological migration of incisors, tooth alignment is completed with the presence of one or more black triangles, due to deficient bone crest height. The distance between the bone crest and the base of the contact point is the main indicator of the complete presence or absence of interdental papillae, even though it is affected by other factors, such as coronal morphology and root distance and divergence.^39^

Reconstruction of the interdental papilla is a challenging and unpredictable problem;^31^ however, the predictability in these cases has been increased by multidisciplinary treatment. This fact must be shared with the patient to assist in decision making regarding the best treatment protocol. Depending on the dental proportions and gingival conditions, orthodontics can act in the repositioning of teeth and closing the diastemas, creating a contact point and reducing the distance between the contact point and the alveolar bone crest by the intrusion movement, and by proximal stripping and space closure.^38^ Other options such as filling with hyaluronic acid,^41^ reshaping with composite resins^31^ or subepithelial connective tissue graft^42^ should be considered in combination or as an alternative to orthodontic options.

During orthodontic movement, a progression of preexisting gingival recessions may occur; therefore, root coverage should be performed after orthodontic treatment.^43^ However, in patients with thin gingival biotype or who require expansion or projection movements, it may be necessary to perform a mucogingival graft before orthodontic treatment.^44^ However, it should be noted that such movements should be avoided in patients with a history of alveolar bone loss.^44^

6) Level 6 - retention

During the retention period, periodontal and orthodontic supervision should be maintained. The interval of consultations can vary for each individual, according to the risk of periodontitis recurrence.^22^ After completion of orthodontic treatment, teeth with insertion loss usually have mobility, and the maintenance of their positions should be considered with caution.

Due to the presence of mobility, many professionals question whether the new positions will be stable, even if they are ideal for maintaining periodontal health after removal of all secondary trauma.^11^ Basically, two primary factors are involved in the balance that determines the final position of the tooth.^45^ These are: 1) the pressure caused by the tongue, lips and cheeks in their rest positions; and 2) forces produced by the metabolic activity in the periodontal ligament. When the periodontium is intact, the unbalanced forces of the lips and tongue are normally compensated by the periodontal ligament. However, when the periodontium is compromised, these forces are no longer counterbalanced, and the teeth begin the migration process. Therefore, since bone loss persists after tooth movement, definitive retention of these teeth must be performed, to preserve their stability in new positions.^11^

If the patient has a clenching or bruxism habit, it is recommended to make an acrylic interocclusal plate to protect the teeth and periodontium from excessive occlusal forces. Definitive retentions in these cases are also at greater risk of fractures and should be reinforced.

There is evidence that periodontal disease cannot be cured, but it can be controlled.^13^ ^,^ ^18^ Therefore, patients with periodontitis must be monitored and controlled throughout their lives by a periodontist.^8^ ^,^ ^11^ ^,^ ^13^ ^,^ ^20^ ^,^ ^21^ ^,^ ^24^ However, when these patients are also submitted to orthodontic treatment, it is essential to maintain follow-up consultations. Periodontal recurrence can further reduce bone insertion levels and generate additional tooth loss.^43^

With such changes, the occlusal balance is altered and should be reestablished. Occlusal adjustments, prosthetic rehabilitation, modifications or extensions of retainers may be necessary, and interocclusal stabilization plates may be indicated. Instability in the periodontal condition can generate occlusal instability,^45^ thus annual or semiannual orthodontic follow-up becomes essential throughout the patient’s life.

## CASE REPORTS

The priorities of orthodontic treatment of a patient with insertion loss are:

- Try to correct or reduce bone defects.
- Improve the esthetic aspect, with benefit to the patient's self-esteem.
- Correct the tooth positioning to facilitate biofilm control by the patient and periodontist.
- Establish a balanced occlusion, with adequate cusp-fossa contacts, without interference or occlusal trauma.
- Obtain the six keys of occlusion, whenever feasible.^1^ ^,^ ^8^ ^-^ ^11^

The biological limits must be considered in these cases,^1^ ^,^ ^11^ and it is often not possible to achieve the ideal occlusion. However, achieving occlusal balance and eliminating interference should always be considered the main objective, since the repair of connective attachment will only be possible after the elimination of occlusal trauma.^17^

It is also important to consider that, in cases of inadequate oral hygiene, smoking, unsatisfactory response to periodontal treatment and uncompensated diabetes mellitus, the treatment may be contraindicated.^40^ Likewise, in cases of advanced bone loss, presence of furcation lesions, intense dental mobility, teeth with thin bone and gingival tissue and root prominence, there is greater risk of recurrence of periodontal disease^40^ and periodontal follow-up during and after orthodontic treatment should be performed at closer intervals.

There is a considerable risk of relapse of periodontal disease, mainly due to the difficult cleaning resulting from the presence of the appliance and orthodontic accessories.^40^ In case of recurrence, the orthodontic therapy must be stopped immediately: passive archwires should be placed and orthodontic forces must be removed, but the appliance does not need to be removed, so that the orthodontist’s work is not lost. Periodontal treatment should be started and prioritized and, as soon as the patient returns to health, the orthodontic treatment should be activated again.^40^

### Case 1 - Patient with Class II malocclusion with increased overjet, marked curve of Spee and pathological migration of incisors

**Figure 2:**
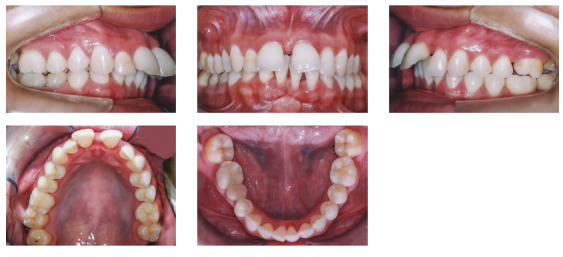
Pre-treatment intraoral photographs of Case 1.

**Figure 3:**
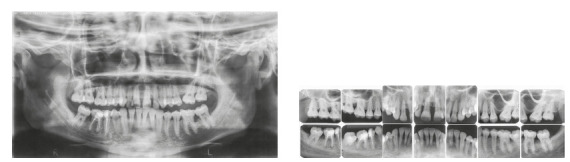
Pre-treatment radiographs of Case 1.

The patient was a young adult (25 years old) presenting Class II division 1 malocclusion, deep overbite, overjet of 7.2 mm, marked lower curve of Spee and pathological migration of the upper incisors, which showed excessive projection, extrusion and diastemas (Fig 2). Chronic periodontal disease also caused significant insertion loss and mobility in the maxillary and mandibular incisors. Significant bone loss was detected at the following sites: 32 (M), 31 (MD), 41 (MD), 42 (MD), 46 (D), 36 (D and bifurcation), 12 (MD), 11 (MD), 21 (MD), 22 (MD), 23 (M), 16 (D), 17 (M), 18 (M) and 27 (M). Marked pneumatization of the maxillary sinus was observed in the mesial aspect of tooth 27, which was mesially inclined (Fig. 3). The periodontal diagnosis identified a variation of 4 to 9 mm in the probing depths and presence of gingival recession in the labial and lingual surfaces of lower incisors, particularly in tooth 31.

**Figure 4:**
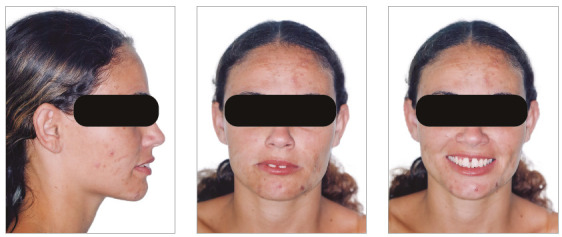
Pre-treatment facial photographs of Case 1.

**Figure 5:**
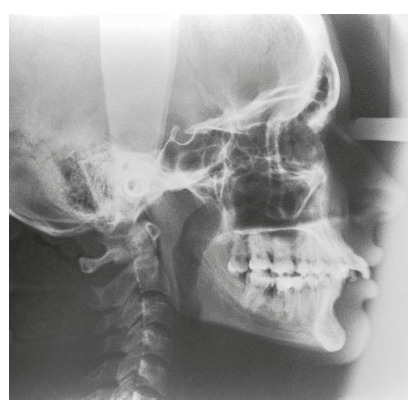
Pre-treatment lateral cephalogram of Case 1.

The chief complaint of the patient was the projection and spacing of maxillary incisors. Facial photographs (Fig. 4) showed a convex facial profile, with incompetent lip sealing and satisfactory chin, with slight deviation to the right. The cephalogram confirmed the extrusion and interposition of maxillary incisors between the lips, marked projection of the maxillary and mandibular incisors, skeletal Class II and brachycephalic pattern (Fig. 5, Tab. 1).

**Table 1:** Cephalometric measurements of Case 1.

Measurements	Pretreatment	Post-treatment	Retention
SNA	86.8°	87.2°	87.2°
SNB	81.9°	83.1°	83.2°
ANB	5.7°	4.1°	3.9°
1.NA	41.4°	23.8°	23.5°
1-NA	10.5 mm	6.0 mm	6.2 mm
1.NB	36.3°	30.1°	30.0°
1-NB	7.2 mm	5.5 mm	5.5 mm
IMPA	107.3°	100.9°	101.2°
Interincisal angle	93.2°	119.0°	118.6°
FMA	18.3°	19.5°	19.2°
SN.Go-Me	25.8°	26.7°	26.2°
PLO (Go-Gn.Ocl)	21.4°	14.9°	15.2°
LAFH (ANS-Me)	69.7 mm	70.0 mm	70.1 mm

As a first step, intensive periodontal treatment was conducted until the disease was controlled and the patient acquired the ability to maintain excellent oral hygiene. After six months of periodontal treatment, the patient was ready to start orthodontic treatment and continued to be supervised quarterly by the periodontist throughout the treatment.

The first orthodontic strategy proposed was to use a modified palatal arch to intrude and retract the upper incisors with indirect anchorage on mini-implants. However, the patient did not accept the use of mini-implants for indirect reinforcement of molar anchorage. Therefore, the alternative was to use a high-pull headgear (HPH) to maintain anchorage and correct the Class II malocclusion. This anchorage option can only be considered after obtaining periodontal health and assessing, with the periodontist, the viability of molars to receive the required load magnitude.

**Figure 6:**
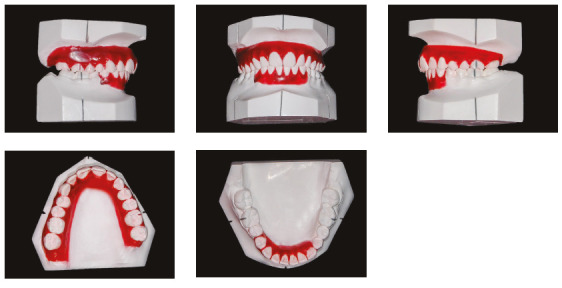
Diagnostic set-up of Case 1.

A diagnostic set-up was performed to assess the viability of the proposed treatment plan (Fig. 6). The set-up showed that acceptable overjet and overbite could be obtained if:

- tooth 18 was extracted;
- tooth 16 was distalized by 4.0 mm;
- teeth 26, 36 and 46 were kept in position, without anchorage loss;
- the lower incisors underwent 2.5 mm of intrusion and 1.0 mm of retraction; and the upper incisors, 2.5 mm of intrusion and 5.0 mm of retraction.

Specific biomechanical planning was necessary to meet the requirements of the amount of movement and the force magnitude used, considering the level of bone insertion of incisors. The set-up demonstrated that, even after intrusion and retraction of upper incisors, there would still be an overjet of 2.5 mm, with black triangles present in the regions between 13-23 and 33-43, especially between 11 and 21. To finalize the reduction of overjet, proximal stripping was required in regions between 13-23 (3.0 mm) and between 33-43 (1.8 mm). The intercanine and intermolar distances remained unchanged, and the midlines were coincident.

### Modified palatal arch

Due to pathological migrations, biomechanical planning for periodontal cases often involves the intrusion and retraction of maxillary incisors with vertical alveolar bone loss - i.e., teeth with a center of resistance positioned more apically, according to the magnitude of bone loss. This phenomenon increases the tendency of uncontrolled inclination,^15^ ^,^ ^18^ which impairs the intrusion and retraction in these cases.

To help the orthodontist to adapt the biomechanics of each orthodontic-periodontal case that requires retraction and intrusion, a modified palatal arch was developed, which allows simultaneous accomplishment of both movements. The design of this arch depends on: number of teeth to be moved; anchorage availability; and amount and direction of movement required. When this arch is used as an aid, the maxillary incisors can be retracted with controlled inclination without the use of orthodontic wires, since the applied force is directed to the center of resistance of each tooth. The direction of retraction can be modified, favoring the movements of intrusion, retraction or retroclination, by the differential positioning of brackets bonded on the palatal surface and connectors soldered to the palatal arch to which NiTi springs will be connected.

In cases with bone loss and pathological migration of incisors (with extrusion, projection and diastemas^18^), palatal brackets should be bonded as cervically as possible, to reduce the tendency of retroclination inherent to the movement, increased by the presence of bone loss.^15^

The magnitude of bone loss of maxillary incisors must be previously assessed on the periapical and cephalometric radiographs to determine the approximate position of the CRes of each tooth that will be moved, according to the amount of remaining bone. The system must be designed so that the force applied by the NiTi springs is as close as possible to the CRes. It is important to highlight that, as the alveolar bone migrates apically, the center of resistance of the tooth also changes, and its distance from the alveolar crest (analyzed on the periapical radiograph) decreases. This distance in teeth with a healthy periodontium is approximately 5.5 mm in relation to the bone crest, and is reduced to 1.6 mm in teeth with bone loss of 8 mm.^19^

When planning the movement, it must be considered that the change in CRes also makes the incisors more prone to inclination than to body retraction, when traditional orthodontic mechanics with continuous archwires are used.^2^ This also occurs when the palatal arch mechanics is applied. Therefore, it is important to bond the palatal brackets as cervically as possible, and that the direction of springs balances the horizontal vector with the amount of remaining bone in teeth to be moved. It is essential to use light forces, not exceeding 10g/tooth, which can be reduced according to the amount of bone loss diagnosed in the moved teeth. The force can be controlled by changing the length of NiTi springs, and not by the position in which they will be connected to the palatal arch, which must be planned according to the biomechanics of movement.

The accomplishment of this treatment protocol also depends on the control of vertical dimension, by the preparation of anchorage to obtain an effective intrusion of incisors. In the clinical Case 1, the anchorage was obtained with passive thick segmented archwires between the canines and the second upper molars, associated with the use of high-pull HPH with light forces for more than 15 hours/day. This modality was selected according to the periodontal condition of the maxillary premolars and molars. In cases of posterior teeth with very deteriorated periodontal conditions or patients who are not willing to cooperate with the use of HPH, direct or indirect anchorage with TADs can be used. Direct anchorage secures the palatal arch to the mini-implants placed on the palate.

### Treatment performed in Case 1

Initially, tooth 18 was extracted to allow distalization. The treatment started with the use of high-pull HPH for at least 15 hours/day, with a force of 150g/side. A standard-edgewise 0.022 x 0.028-in appliance was bonded only to teeth 23-28 and 13-17; and a 0.017 x 0.025-in passive segmented archwire was used to complete the anchorage system. The intrusion was planned with a modified palatal arch placed in the same period, cemented in teeth 17 and 28.

**Figure 7:**
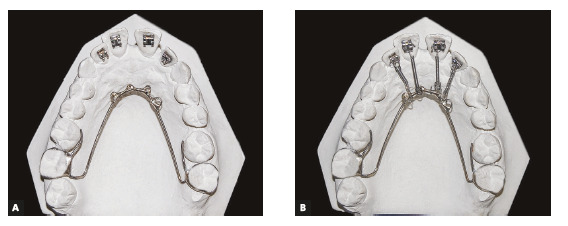
A) Modified palatal arch. B) Palatal arch with activation.

This palatal arch had compressed nickel-titanium (NiTi) springs tied to standard edgewise brackets, which were bonded as cervically as possible to the palatal surface of upper incisors (Fig. 7) to direct the force as close as possible to the center of resistance of incisors, to perform the intrusion and retraction simultaneously. Ten days before the onset of intrusion with the modified palatal arch, periodontal surgery was performed with scaling and root planing.^13^ After surgery, orthodontic intrusion became a more reliable therapeutic treatment, which improved the esthetics, periodontal health and bone levels of intruded teeth.^13^

**Figure 8:**
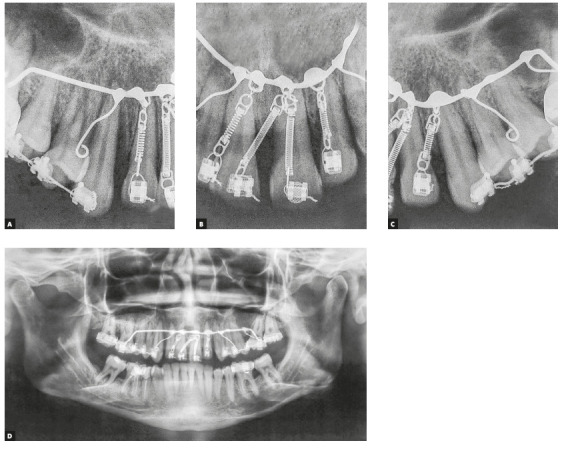
A-C) Periapical radiographs of the activated palatal arch in the patient. D) Panoramic radiograph of the activated palatal arch in the patient.

**Figure 9:**
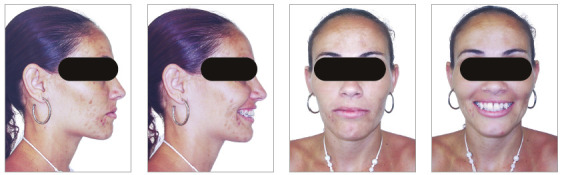
Extraoral photographs after eight months of intrusion.

The retraction and intrusion of these teeth were initiated with a 12-mm NiTi compressed spring tied to each of the four incisors, with the line of force action passing close to the center of resistance, displaced apically, due to the bone loss of these teeth (Fig. 8), which generated a force of 10g/tooth. During this period, orthodontic maintenance was performed at every six weeks, and the springs were progressively replaced by smaller springs, until reaching the 5-mm spring, maintaining the same force of 10g per tooth. The intrusion and retraction of anterior teeth with this system were performed for eight months. The interincisal angle gradually decreased after the first two months of retraction. The spaces were closed, while the posterior occlusal relationship was maintained (Fig. 9). At that moment, it was possible to observe the black triangles and plan the proximal stripping after joint evaluation with the periodontist: the incisors showed a subtle improvement in the clinical insertion levels (with reduction of probing depth), absence of inflammation and bleeding sites, and mobility compatible with the movement performed.

Subsequently, the palatal brackets were removed, and a standard Edgewise 0.022 x 0.028-in appliance was bonded to both arches. The HPH was maintained to achieve the Class I molar relationship. The initial 0.012-in stainless steel wire was used in the upper arch. For uprighting of the third mandibular molars, a cantilever was made with 0.017 x 0.025-in beta titanium wire on the 0.019 x 0.025-in stainless steel passive base archwire.^8^

When there is a need to level the curve of Spee in patients with vertical bone loss, the intrusion of lower incisors should be planned considering the tendency of increased buccal projection of these teeth, due to the change in the CRes and the need for reduced strength. Therefore, continuous archwire mechanics^2^ should be avoided. Whenever the minimum projection is desired, it is recommended to use the three-piece intrusion mechanics, recommended by Burstone,^46^ fitting the beta titanium cantilevers (0.017 x 0.025-in) in the region compatible with the distal surface of 33 and 43 in the passive base archwire (stainless steel 0.019 x 0.025-in).^46^ The force magnitude can be controlled by checking the force applied by the cantilevers on the base archwire, to use a force compatible with the level of bone loss in the incisors.

In this case, a magnitude of 5mg per tooth was calculated (20mg/side, which was measured and maintained with each activation). Then, the alignment and leveling of the lower arch was started with a 0.014 x 0.025-in nickel-titanium continuous archwire. Sequentially, thicker rectangular stainless steel wires were used to level the mandibular and maxillary arches, and the crossbite was corrected with symmetrical and coordinated archwires.

To correct the black spaces and normalize the remaining overjet, proximal stripping of 13-23 (3.4 mm) and 33-43 (2.0 mm) was necessary. A 0.019 x 0.025-in stainless steel retraction archwire with T-shaped spring was used to avoid the uncontrolled inclination of incisors during space closure as much as possible. The treatment was ended with ideal coordinated 0.019 x 0.025-in archwires.

After 19 months of treatment, a stable occlusion was obtained. Root parallelism was confirmed on the panoramic radiograph, and all appliances were removed. During orthodontic treatment, periodontal control was performed by a periodontist at every three months. The retention was made with 0.018-in stainless steel wire, bonded from 3-3 in the maxillary and mandibular arches, splinting the anterior teeth.

**Figure 10:**
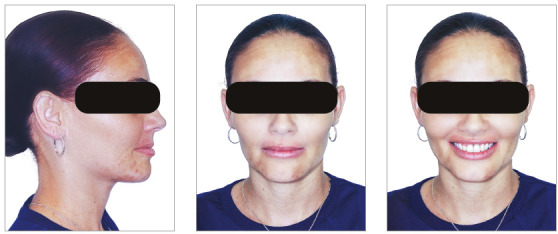
Posttreatment facial photographs of Case 1.

**Figure 11:**
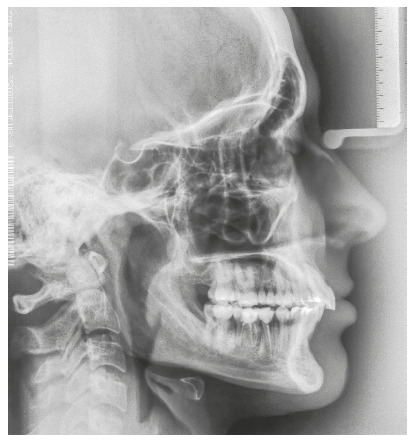
Posttreatment lateral cephalogram of Case 1.

**Figure 12:**
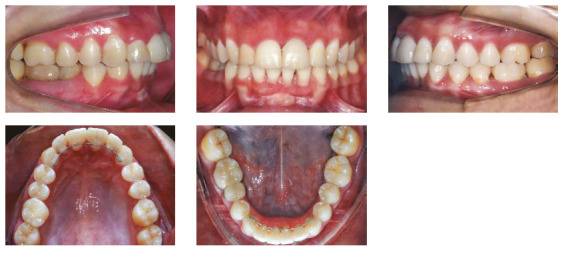
Posttreatment intraoral photographs of Case 1.

**Figure 13:**
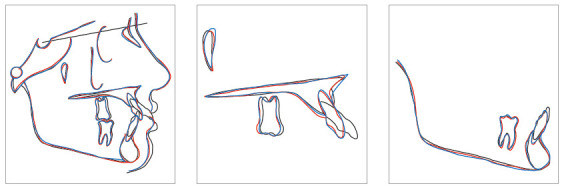
Cephalometric superimpositions of Case 1.

Posttreatment facial photographs showed that there was a reduction in the protrusion of incisors, and a well-balanced face was obtained, due to the upper lip retraction (Fig. 10). The cephalometric analysis (Fig. 11, Tab. 1) showed a small increase in the FMA angle, reduction of ANB from 5.7° to 4.1° and intrusion and retraction of maxillary anterior teeth. The mandibular incisors were intruded and lingually inclined, and the interincisal angle was reduced to the normal pattern (Fig. 12). The cephalometric superimposition confirmed that there was bodily retraction, retroclination and intrusion of 3.2 mm of the maxillary anterior teeth (Fig. 13). The maxillary posterior teeth were distalized with minimal extrusion (almost null), due to the vertical control of the HPH. Thus, the lower facial height was maintained at an adequate proportion, without chin retrusion, which resulted in a harmonious facial result (Fig. 10).

**Figure 14:**
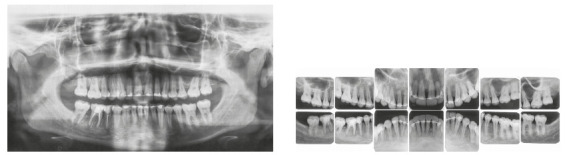
Posttreatment radiographs of Case 1.

The treatment results were within acceptable limits, and the patient was satisfied. Periapical and panoramic radiographs (Fig. 14) showed good root parallelism and no noticeable root resorption. The radiographs showed reduction in the dimensions of the angular infrabony defects in the regions of maxillary and mandibular molars and in the maxillary incisors.

**Figure 15:**
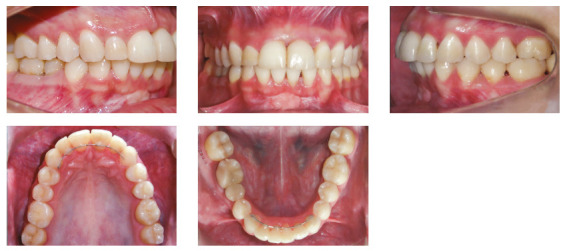
Intraoral retention photographs of Case 1.

**Figure 16:**
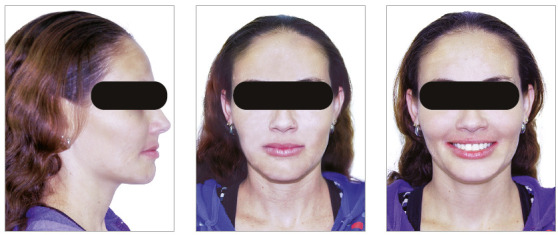
Extraoral retention photographs of Case 1.

**Figure 17:**
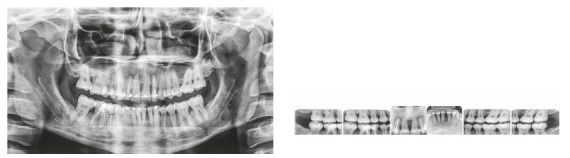
Retention radiographs of Case 1.

**Figure 18:**
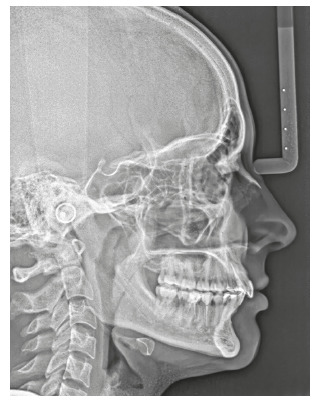
Retention lateral cephalogram of Case 1.

During active orthodontic treatment, probing depths and bone levels in the anterior segment, radiographically assessed, were maintained at the levels reached after initial periodontal treatment. The occlusal stability of this case was maintained with semiannual periodontal consultations and semiannual maintenance of the fixed retainers, and can be observed 24 months after treatment completion, both from a clinical (Fig. 15 and 16) and radiographic (Fig. 17 and 18, Tab. 1) standpoint.

### Case 2 - Pathological migration of upper incisors and Class II

**Figure 19:**
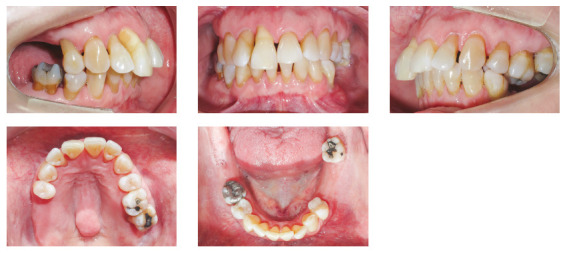
Initial intraoral photographs of Case 2.

The patient was an elderly woman (62 years old), with Class II division 1 malocclusion, deep overbite, overjet of 6.8 mm, moderate lower curve of Spee. She also presented pathological migration of maxillary incisors (projected, extruded and with diastemas), especially in tooth 11 (Fig. 19).

**Figure 20:**
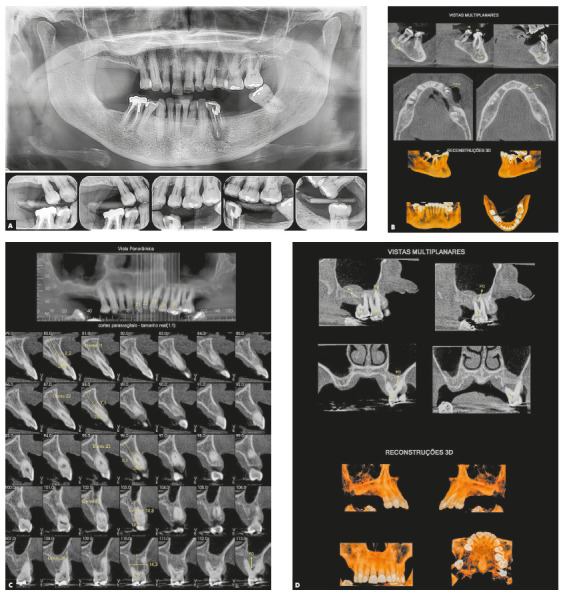
A) Initial radiographs of Case 2. B-D) Initial tomographic sections of areas of interest of Case 2.

Chronic periodontitis caused significant insertion loss and mobility in the maxillary and mandibular incisors, in addition to extraction of teeth 16, 17, 47, 35 and 36. Significant insertion loss was detected in the following areas: 34 (M), 33 (MD), 32 (MD), 31 (MD), 41 (MD), 42 (MD), 43 (M), 48 (M and bifurcation), 15 (MD), 13 (M), 12 (MD), 11 (MD), 21 (MD), 22 (MD), 23 (M), 26 (D and furcation) and 27 (MD and furcation with large extension). In addition, tooth 34 was fractured and tooth 46 had bone rarefaction in the healing phase (Fig. 20). The periodontal diagnosis detected probing depths of 3-9 mm and the presence of gingival recessions on the buccal and lingual surfaces of all present teeth, most significantly in teeth 11 and 12.

**Figure 21:**
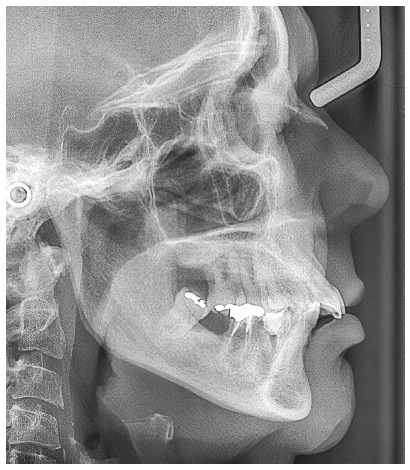
Initial lateral cephalogram of Case 2.

The cephalogram confirmed the extrusion of maxillart incisors, marked projection of the maxillary and mandibular incisors, skeletal Class II and dolichocephalic pattern (Fig. 21, Tab. 2).

**Table 2:** Cephalometric measurements of Case 2.

Measurements	Pretreatment	Post-treatment
SNA	83.9°	84.1°
SNB	78.1°	80.0°
ANB	5.8°	4.1°
1.NA	30.3°	25.9°
1-NA	6.4 mm	4.5 mm
1.NB	31.6°	30.2°
1-NB	6.1 mm	6.3 mm
IMPA	95.8°	95.0°
Interincisal angle	113.4°	118.9°
FMA	29.7°	26.6°
SN.Go-Me	36.9°	35.4°
PLO (Go-Gn.Ocl)	21.8°	18.1°
LAFH (ANS-Me)	71.7mm	70.3mm

**Figure 22:**
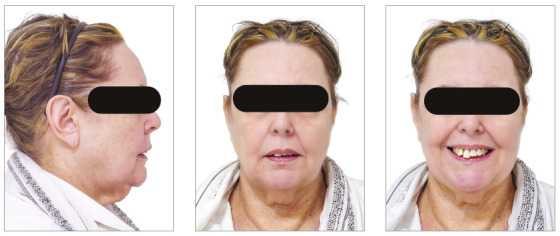
Initial extraoral photographs of Case 2.

The chief complaints of the patient were extrusion of tooth 11, interincisal diastemas and missing teeth. The facial photographs (Fig. 22) presented a convex facial profile, with incompetent lip sealing and satisfactory chin, with slight deviation to the right.

**Figure 23:**
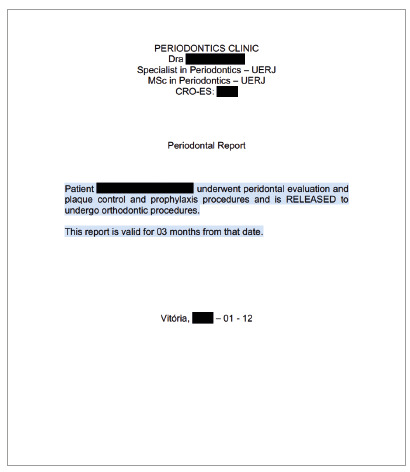
Example of a periodontal report.

As a first step, intensive periodontal treatment was performed until the disease was controlled and the patient acquired the ability to maintain good oral hygiene, which was supervised monthly by the periodontist throughout the treatment. Subsequently, due to the significant improvement in periodontal indices and in oral hygiene, the consultations became quarterly. It is important to note that the frequency of periodontal consultations can be changed, according to the compliance with hygiene and tissue response of the patient throughout the orthodontic treatment. It is recommended to request a report from the periodontist confirming the release for orthodontic movement, as well as its validity (Fig. 23).

**Figure 24:**
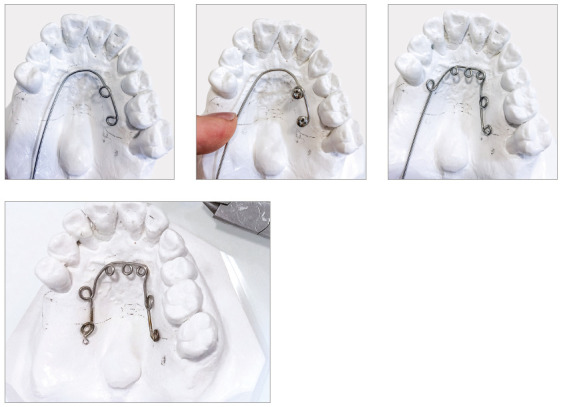
Modified palatal arch with direct anchorage in TADs.

Orthodontic planning in this case also used the modified palatal arch with direct anchorage on TADs to intrude and retract the maxillary incisors. The palatal arch was made to be fixed to four mini-screws (Fig. 24). We opted for the use of TADs because of the impossibility of using the posterior maxillary teeth as anchorage, due to marked insertion loss and absence of tooth 16.

Due to the marked loss of insertion, it was decided to extract teeth 35 and 27. Dental implants were planned in the regions of 16, 34 and 35. Since the implant placement for tooth 16 required bone grafting, it was not initially considered as an anchorage option. The implant of tooth 16 was later used as anchorage for Class II correction, combined with an extra-alveolar mini-implant on the left side. However, due to the patient's age and extensive bone loss, this treatment had significant biological limitations. After correcting the pathological migration of maxillary incisors, additional retraction was necessary, whose planning was performed in combination with the periodontist.

The interincisal black spaces, already present between the four incisors, would be increased after correcting the extrusion of tooth 11. To avoid this, proximal stripping between 11-21 (0.3 mm) and between 11-12 and 21-22 (0.2 mm) was planned. The feasibility of additional retraction of incisors would be reevaluated after the correction of migrations, conditioned to the biological response of these teeth. The remaining black spaces could be closed with direct composite resin restorations. However, reshaping with resin should be limited due to the contraindication of creating biofilm retention sites. Therefore, the patient was advised that black spaces would be reduced, but they might not be completely solved. The treatment limitations should be clarified at the planning stage, to prevent the patient from creating unrealistic expectations about the results, since biological limitations in the treatment of periodontal patients are frequent.

**Figure 25:**
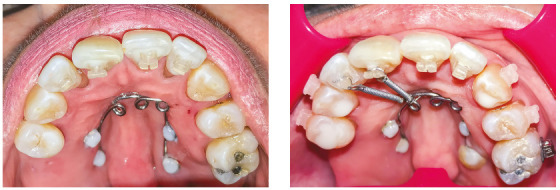
Modified palatal arch in activation, in Case 2.

The retraction and intrusion were initiated with a 12-mm compressed NiTi spring tied to the four maxillary incisors. The line of action of the applied force was close to the center of resistance of incisors (apically displaced due to bone loss) and generated a force of 10 g/tooth in teeth 21, and 22 and force of 5 g/tooth in teeth 11 and 12. As the occlusal contact of tooth 21 generated a fremitus, a posterior occlusal stop was made to de-occlude this tooth, eliminating the secondary occlusal trauma. At first, only the intrusion of teeth 21 and 22 was initiated (Fig. 25).

When teeth 21 and 22 were leveled with teeth 11 and 12, the intrusion movement became simultaneous in the four incisors. Orthodontic maintenance consultations were performed at every six weeks. The springs were progressively reduced until reaching the 5-mm spring, maintaining the same force intensity.

The intrusion and anterior retraction were performed for six months. Then, the spaces were closed, maintaining the occlusal relationship of posterior teeth. Since tooth 27 showed relapse of periodontal inflammation (with worsening of insertion loss on the mesial and distal surfaces), it was indicated for extraction. At that time, the lingual brackets were removed, and fixed 0.022 x 0.028-in Roth prescription orthodontic brackets were bonded to both arches.

At this stage, the implant in the region of tooth 16 was placed in Class I relationship, after checking the quality of bone grafting performed at the site. Implants in regions of teeth 35 and 36 had been previously placed. Together with the periodontist, a consensus was reached that Class II correction would be feasible only on the right side, since additional retraction of the incisors would be possible; however, the movement of tooth 26 was contraindicated.

Therefore, due to the biological limitation present, a Class I relationship was established on the right side and Class II was maintained on the left side. Proximal stripping was performed to minimize the black triangles, keeping the coincident midlines. In the laterality guides, a group function was established on the left side and canine guidance on the right side. The stripping spaces were closed with a 0.019 x 0.025-in stainless steel retraction archwire, with a T-shaped spring, to control the inclination of incisors. The treatment was completed with ideal coordinated 0.019 x 0.025-in archwires.

After 24 months of treatment, a stable occlusion was obtained. Root parallelism was confirmed on the panoramic radiograph, and the appliances were removed. The patient chose not to reshape the remaining black triangles with composite resins, as they were not visible in the smile and because she did not want to impair her hygiene. During the treatment period, periodontal control was performed by a periodontist at every three months. The retention was made with 0.018-in stainless steel wire, bonded on 3-3 in the maxillary and mandibular arches, splinting the anterior teeth.

**Figure 26:**
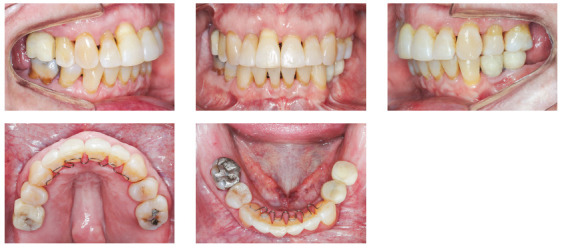
Final intraoral photographs of Case 2.

**Figure 27:**
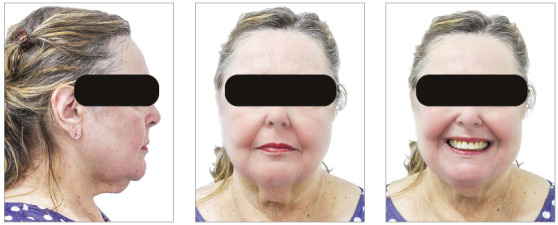
Final extraoral photographs of Case 2.

The posttreatment intraoral photographs showed leveling of incisors, reduction of extrusion and protrusion, achieving adequate overbite and overjet and rehabilitation of edentulous areas (Fig. 26). The face was well balanced, due to the intrusion and retraction of incisors, with consequent upper lip retraction and obtaining passive lip sealing (Fig. 27).

**Figure 28:**
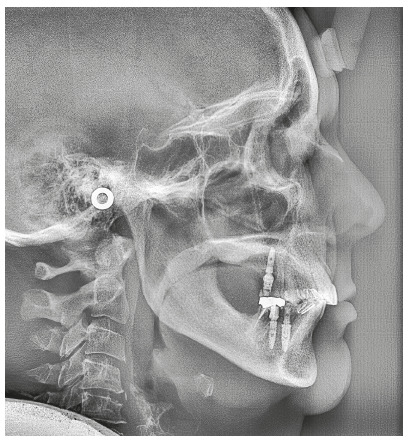
Final lateral cephalogram of Case 2.

**Figure 29:**
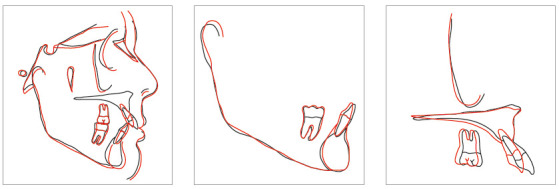
Cephalometric superimposition of Case 2.

The cephalometric analysis (Fig. 28, Tab. 2) showed a small increase in the FMA angle, reduction in ANB (from 5.7° to 4.1°), intrusion and retraction of maxillary incisors. Analysis of the cephalometric superimposition confirmed that there was bodily retraction and retroclination of upper incisors, and an intrusion of 3.8 mm from the apex of tooth 11 (Fig. 29). The treatment results were clinically acceptable, and the patient was satisfied.

**Figure 30:**
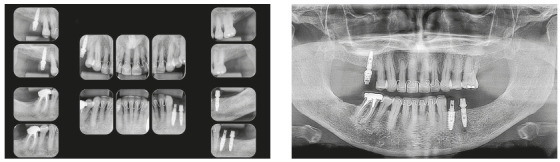
Final radiographs of Case 2.

**Figure 31:**
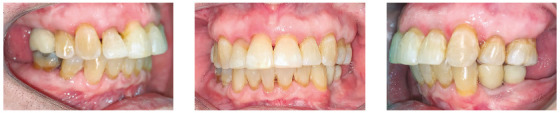
Intraoral photographs of the retention phase of Case 2.

**Figure 32:**
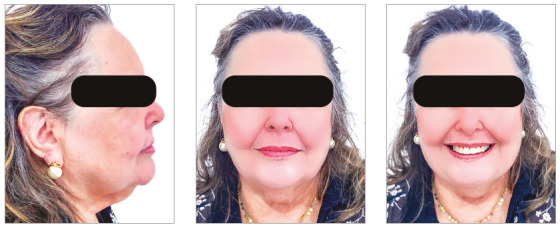
Extraoral photographs of the retention phase of Case 2.

The periapical and panoramic radiographs (Fig. 30) showed good root parallelism, absence of root resorption and reduction of angular infrabony defects in the regions of maxillary and mandibular molars and maxillary incisors. The stability of the case was maintained with periodontal consultations at every four months, with orthodontic maintenance of the fixed retention bars, and can be verified 12 months after treatment completion (Figs 31 and 32).

### Case 3 - atypical pathological migration of mandibular incisors

**Figure 33:**
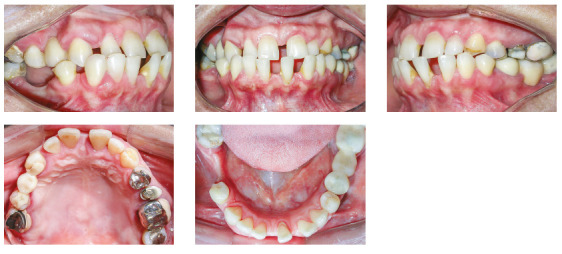
Initial intraoral photographs of Case 3.

The patient was an adult (50 years old), with Class I malocclusion, anterior crossbite, negative protrusion of -2.3 mm, bilateral posterior crossbite and diastemas between the maxillary and mandibular incisors. Significant pathological migration in incisors generated 5.6 mm of diastema in the mandibular arch and 2.9 mm in the maxillary arch. The mandibular arch still had a marked curve of Spee and the maxillary arch presented a reverse curve of Spee (Fig. 33). The patient also had nocturnal bruxism, with significant occlusal wear, especially in the canines.

**Figure 34:**
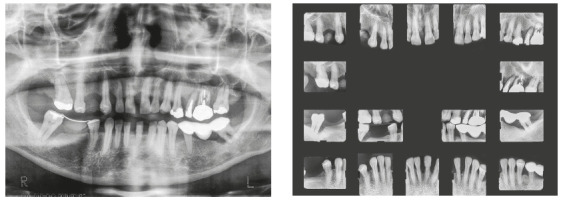
Initial radiographs of Case 3.

Chronic periodontal disease also caused insertion loss, mobility in all incisors and loss of teeth 14, 17, 36, 45 and 46. Significant bone loss was detected in the following areas: 31 (MD), 41 (MD), 34 (D), 35 (MD), 37 (M), 47 (M), 11 (MD), 12 (D), 13 (M), 15 (M), 21 (M), 24 (D), 25 (MD), 26 (MD) and 27 (M) (Fig. 34). The probing depths varied from 4 to 10 mm. Gingival recessions were observed on the labial and lingual surfaces of lower incisors, particularly in tooth 31.

**Figure 35:**
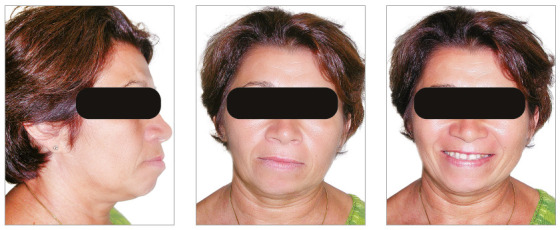
Initial extraoral photographs of Case 3.

**Figure 36:**
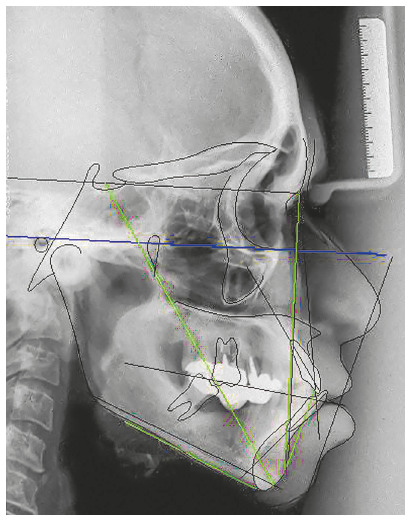
Initial lateral cephalogram of Case 3.

The chief complaints of the patient were negative overjet, missing teeth and lower and upper interincisal diastemas. In facial photographs (Fig. 35), a convex facial profile was observed, with competent lips and satisfactory chin. The cephalogram confirmed the marked projection and protraction of the mandibular incisors, skeletal Class II and brachycephalic pattern (Fig. 36, Tab. 3).

**Table 3:** Cephalometric measurements of Case 3.

Measurements	Pretreatment	Post-treatment
SNA	83.6°	82.2°
SNB	77.9°	80.1°
ANB	6.7°	2.1°
1.NA	25.5°	28.8°
1-NA	1.9 mm	4.7 mm
1.NB	42.9°	30.0°
1-NB	13.2 mm	5.6 mm
IMPA	108.4°	96.5°
Interincisal angle	104.5°	118.5°
FMA	22.2°	19.5°
SN.Go-Me	23.6°	22.7°
PLO (Go-Gn-Ocl)	17.9°	20.5°
LAFH (ANS-Me)	65.2 mm	66.1 mm

As a first step, intensive periodontal treatment was performed until periodontitis was controlled and the patient acquired the ability to maintain good oral hygiene. The initial planning was to perform periodontal control on a quarterly basis; however, during the intrusion of mandibular incisors, the frequency of these consultations became monthly, due to the large accumulation of calculus in this region, due to the patient’s difficulty with oral hygiene. During the intrusion movement, this control is even more important, due to the risk of displacing supragingival biofilm to the subgingival region, worsening the formation of infrabony periodontal pockets.^5^ In some cases, periodontal consultations may be monthly until completion of the intrusion movement, so that it is possible to maintain and even improve the health of the reduced periodontium.^20^ ^,^ ^21^

The diagnostic set-up showed that the correction of overjet required intrusion of 3.6 mm of the mandibular incisors. Since these teeth had vertical alveolar bone loss, the three-piece intrusion mechanics of Burstone^46^ associated with dental implants was planned, which were immediately placed to provide adequate anchorage for the cantilevers of this technique.

**Figure 37:**
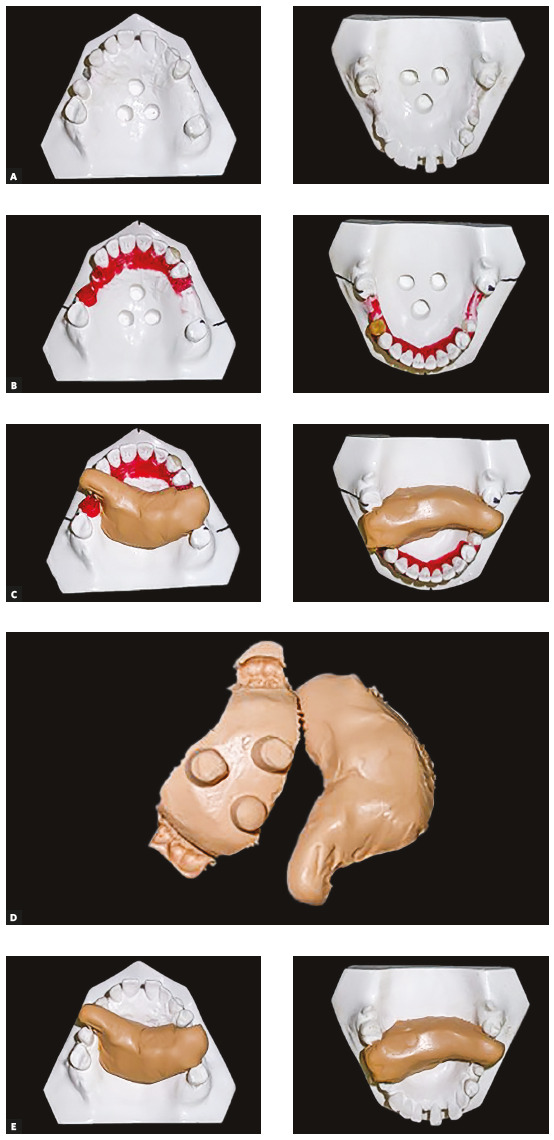
A) Retentions made in the initial model of the patient. B) *S*et-up made in the duplicated model with retentions*.* C-E) Making and transferring the silicone guides.

Since it was planned to place dental implants in the regions of teeth 14, 25, 26, 36, 46 and 47, and to extract the tooth 37 (condemned by the periodontist), the diagnostic set-up was used to create a guide to the surgical positioning of implants using addition silicone. To make this guide, it is necessary to make retentions in the initial study model of the patient (Fig. 37A) and then duplicate it with guides for the set-up preparation (Fig. 37B). After completing the set-up, the crowns of future implants are positioned on the model and then the silicone guide is made, which can be transferred to the patient's initial study model (Fig. 37C, D and E).

A standard Edgewise 0.022 x 0.028-in appliance was bonded to the maxillary and mandibular dental arches. The initial alignment and leveling was performed with multiloop 0.012-in stainless steel archwire with tieback, to avoid the projection of incisors. Immediately after the initial alignment, the intrusion mechanics was started, with a passive 0.019 x 0.025-in stainless steel base archwire in the region of mandibular incisors with a projection of 3 mm to the distal surface of canines. This planning allowed the line of force action to pass as close as possible to the center of resistance of the mandibular incisors block (displaced distally to the canines, due to bone loss).^45^ The cantilevers were made from 0.017 x 0.025-in beta titanium archwire, activated at every five weeks, applying an intrusion force of 20 mg per side (5mg on each tooth) for six months.

**Figure 38:**
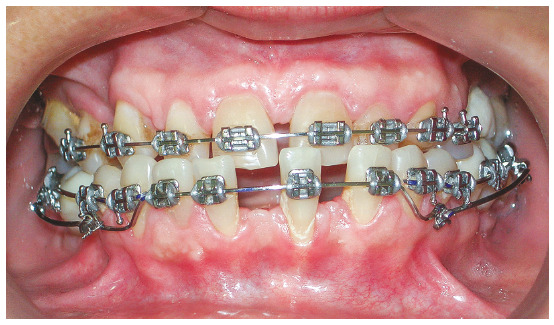
Deficient hygiene during the intrusion movement.

During the six months of intrusion, the patient attended monthly consultations with the periodontist for scaling and root planing, due to poor hygiene in the region (Fig. 38). In fact, during the intrusion movement, monthly periodontal control can be indicated.^40^ After completion of intrusion, consultations with the periodontist became quarterly and a 0.016-in lower multiloop stainless steel archwire with tieback was used to align and level the teeth. A sequence of progressively thicker archwires was used to obtain proper alignment and leveling, until the use of a 0.019 x 0.025-in stainless steel archwire with T-spring to close the maxillary and mandibular spaces.

After the spaces were closed, it was decided to maintain the remaining black triangles in the mandibular arch, to avoid proximal stripping on the incisors, due to the unfavorable anatomy, and to minimize their movement. The patient chose not to reshape the region with composite resins. The treatment was completed with ideal coordinated 0.019 x 0.025-in stainless steel archwires.

**Figure 39:**
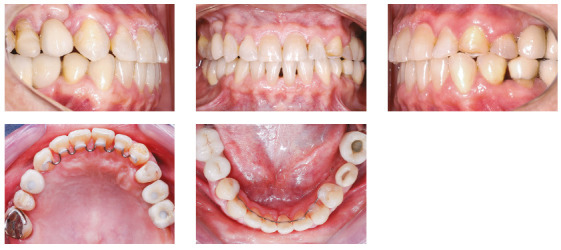
Final intraoral photographs of Case 3.

After 30 months of treatment, a stable occlusion was obtained. Root parallelism was confirmed on the panoramic radiograph, and the appliances were removed. The orthodontic retainer splinted the anterior teeth with 0.020-in stainless steel wire bonded on 4-4 in the mandibular arch and 0.018-in stainless steel wire bonded on 3-3 in the maxillary arch. A bite plate was installed to distribute the forces equally in the posterior region and protect the anterior region, leaving it without contact. The posttreatment intraoral photographs show the leveling of maxillary and mandibular incisors, and the closure of diastemas, as well as reduction of extrusion and protrusion of mandibular incisors, obtaining adequate overbite and overjet, and the prosthetic rehabilitation of edentulous areas (Fig. 39).

**Figure 40:**
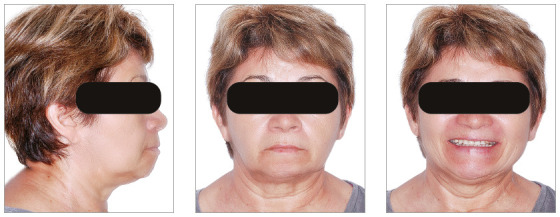
Final extraoral photographs of Case 3.

**Figure 41:**
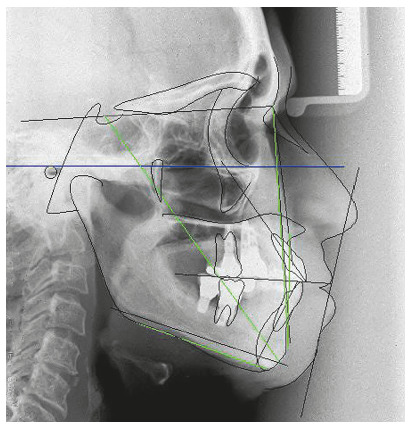
Final lateral cephalogram of Case 3.

**Figure 42:**
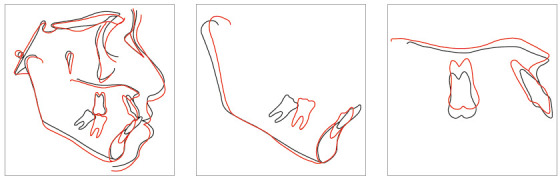
Cephalometric superimposition of Case 3.

Posttreatment facial photographs showed reduced incisor protrusion, and a youthful and well-balanced face, due to lip retraction (Fig. 40). The cephalometric analysis (Tab. 3, Fig. 41) showed a small increase in the FMA angle, reduction in ANB (from 5.7° to 4.1°), intrusion and retraction of maxillary incisors. The mandibular incisors were intruded and lingually inclined, with improvement in the interincisal angle, which was reduced to normal values. The cephalometric superimposition confirmed the bodily retraction, retroclination and intrusion of 3.2 mm of the mandibular incisor apices (Fig. 42).

**Figure 43:**
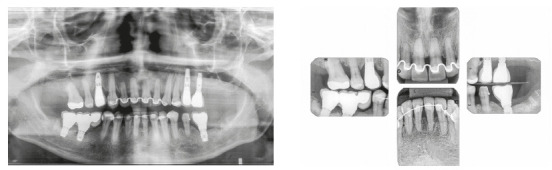
Final radiographs of Case 3.

The treatment results were within acceptable limits, and the patient was satisfied. Periapical and panoramic radiographs (Fig. 43) showed good root parallelism and mild root resorption in the maxillary and mandibular incisors. The radiographs showed reduction in the dimensions of angular infrabony defects in the regions of maxillary and mandibular incisors.

**Figure 44:**
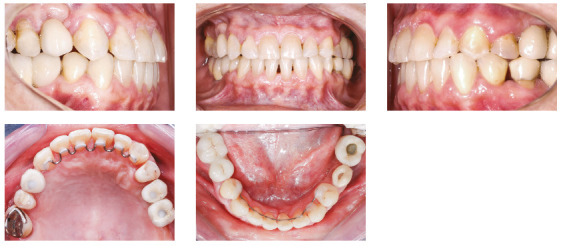
Intraoral retention photographs of Case 3.

**Figure 45:**
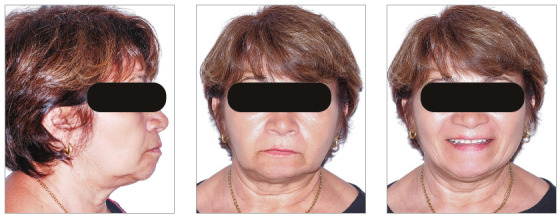
Extraoral retention photographs of Case 3.

**Figure 46:**
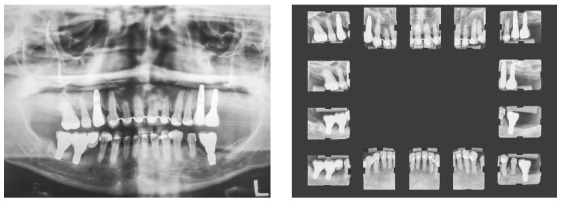
Retention radiographs of Case 3.

During the active orthodontic treatment, probing depths and bone levels in the anterior region (radiographically assessed) were maintained at the levels reached after initial periodontal treatment. The stability of this case was maintained with semiannual periodontal consultations and orthodontic maintenance of retainers and the bite plate, which can be clinically (Fig. 44 and 45) and radiographically (Fig. 46) observed 30 months after treatment completion.

### Case 4 - Alveolar bone loss in maxillary incisors in biprotrusive patient

**Figure 47:**
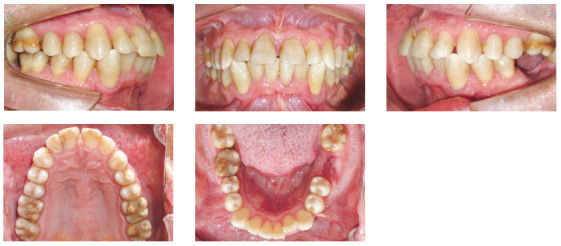
Initial intraoral photographs of Case 4.

The patient was an elderly man (68 years), with Class I malocclusion, biprotrusive incisors, overjet of 4.2 mm, diastemas between the maxillary central incisors and moderate anterior lower crowding (-5.1 mm). Pathological migration generated significant protrusion in the maxillary incisors and 2.3 mm of upper midline diastema. The mandibular arch had a marked curve of Spee and the maxillary arch had a reverse curve of Spee (Fig. 47).

**Figure 48:**
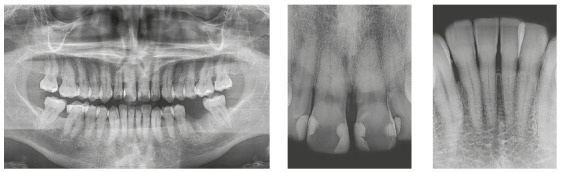
Initial radiographs of Case 4.

Chronic periodontitis caused mobility and significant insertion loss in the maxillary incisors at the following sites: 11 (MD), 12 (M), 21 (MD), 22 (M). The periodontal diagnosis showed that the probing depths varied from 4 to 7 mm and that gingival recession was observed on the labial surfaces of maxillary central incisors and mandibular canines. Tooth 36, which had a bulky metallic pin, was condemned due to the presence of root fracture (Fig. 48). The patient also had daytime tooth clenching (centric bruxism).

**Figure 49:**
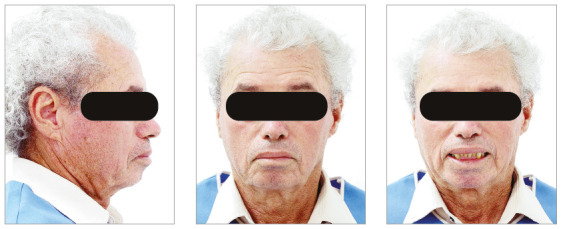
Initial extraoral photographs of Case 4.

**Figure 50:**
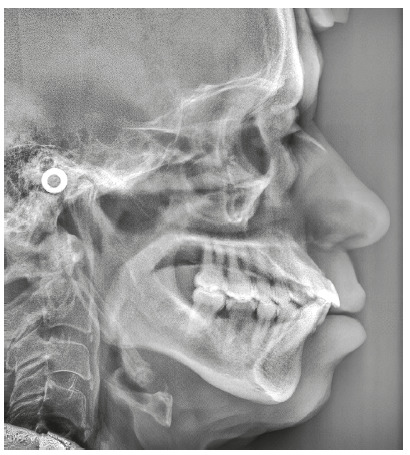
Initial lateral cephalogram of Case 4.

The chief complaints of the patient were the midline diastema and the difficulty in lip sealing. Facial photographs (Fig. 49) showed a convex facial profile, with competent lips and a satisfactory chin. The cephalogram confirmed biprotrusion of incisors, skeletal Class I and a brachycephalic pattern (Fig. 50, Tab. 4).

**Table 4:** Cephalometric measurements of Case 4.

Measurements	Pretreatment	Post-treatment
SNA	85.0°	85.9°
SNB	82.9°	83.9°
ANB	2.1°	2.0°
1.NA	39.2°	29.8°
1-NA	11.2 mm	7.4 mm
1.NB	40.7°	36.0°
1-NB	10.2 mm	8.8 mm
IMPA	108.4°	103.5°
Interincisal angle	107.7°	100.5°
FMA	19.8°	20.5°
SN.Go-Me	30.1°	29.8°
PLO (Go-Gn-Ocl)	18.0°	15.5°
LAFH (ANS-Me)	69.3 mm	69.1 mm

As a first step, intensive periodontal treatment was performed until the disease was controlled and the patient was trained to maintain excellent oral hygiene. Periodontal control was performed quarterly throughout the treatment and the patient cooperated with a good standard of hygiene.

After this step, tooth 36 was replaced by a dental implant with a provisional crown. Then, a 0.022 x 0.028-in Roth prescription fixed orthodontic appliance was bonded to both arches. The space to dissolve the anterior lower crowding was obtained by proximal stripping, performed since insertion of the first archwire, to avoid further projection of the mandibular incisors. Alignment was performed with 0.012-in braided stainless steel archwires with tied omegas and a step up fold in the incisors region, to avoid the use of continuous leveling. The alignment evolved with 0.014-in, 0.016-in and 0.018-in braided stainless steel archwires, always with tied omegas and the step up in the incisors region.

After alignment, the intrusion was planned using three-piece intrusion mechanics of Burstone^46^ in the mandibular arch, to correct the marked curve of Spee and provide sufficient overjet to enable the retraction of maxillary teeth (to subsequently correct the protrusion). The mandibular molars did not present vertical bone loss and thus were considered an adequate anchorage for the use of cantilevers in this mechanics.

The intrusion mechanics was initiated by placing the 0.019 x 0.025-in passive stainless steel base archwire in the region of mandibular incisors, with a projection that coincided with the distal surface of canines, so that the line of force action passed as close as possible to the center of resistance of the mandibular incisors block. Since there was no bone loss in the mandibular incisors, it was not necessary to make a entension beyond the distal of canines.^45^ The 0.017 x 0.025-in beta titanium wire cantilevers were activated at every five weeks and applied an intrusion force of 40 mg per side (10 mg on each tooth) for five months. After completion of intrusion, a 0.017 x 0.025-in lower braided steel archwire with tied omegas was used to finalize the alignment and leveling. In the maxillary arch, alignment and leveling were performed concomitantly and also used progressively thicker braided and steel wires with tied omegas.

**Figure 51:**
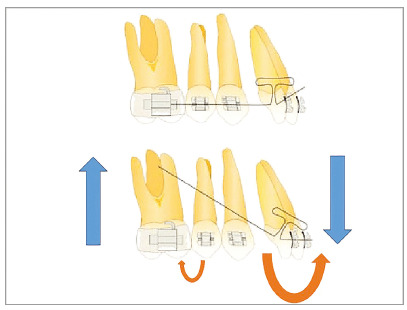
Illustration of the T-shaped spring and bend, accentuating the Gable effect on the T-spring, to prevent the extrusion of incisors during retraction.

The retraction of maxillary incisors was performed with a 0.019 x 0.025-in stainless steel retraction archwire with a T-spring and a bend accentuating the Gable effect on the T-spring to prevent the extrusion of incisors during retraction (Fig. 51). This closed the midline diastema and also the spaces of proximal stripping of 0.2 mm/surface on all anterior teeth, between the mesial aspects of teeth 14 and 24. Since there was a need to correct the excessive protrusion of maxillary incisors and the upper reverse curve of Spee, it would be possible to consider the retraction with continuous wires; however, the presence of marked alveolar bone loss always contraindicates this type of mechanics. Due to the apical migration of the CRes, if retraction with continuous archwires was used, there would be uncontrolled inclination from the apex of incisors to the buccal side, the malocclusion would not be corrected and this might generate side effects, such as root resorption. Due to the proximal stripping made, after the spaces were closed, there were no remaining black triangles. The treatment was completed with ideal 0.019 x 0.025-in coordinated archwires.

**Figure 52:**
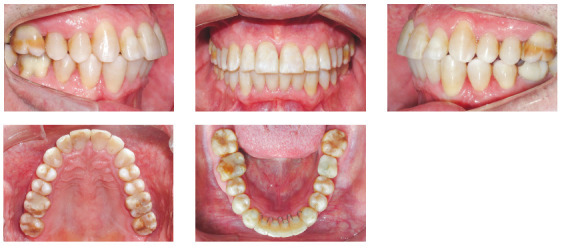
Final intraoral photographs of Case 4.

After 22 months of treatment, a stable occlusion was obtained. Root parallelism was confirmed on the panoramic radiograph, and the devices were removed. The 0.018-in stainless steel wire retainer was bonded on 3-3 to the mandibular arch, complemented by a removable rigid acetate plate for daytime use, which also aided in clenching control, as a reminder for the patient. Due to centric bruxism, a nighttime bite plate was also installed, which equally distributed the forces in the posterior region and protected the anterior region, leaving it without contact. The posttreatment intraoral photographs showed that the leveling of incisors, closure of the diastema, rehabilitation of the edentulous area and correction of biprotrusion, overbite and overjet were achieved (Fig. 52).

**Figure 53:**
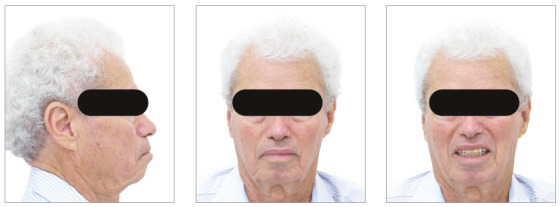
Final extraoral photographs of Case 4.

**Figure 54:**
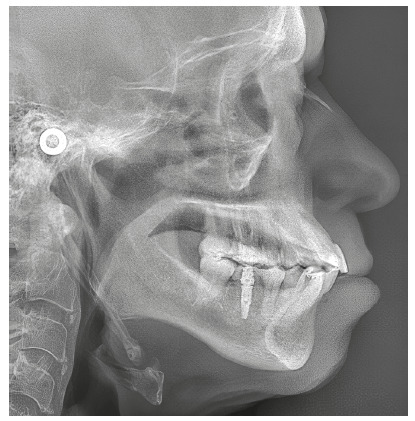
Final lateral cephalogram of Case 4.

**Figure 55:**
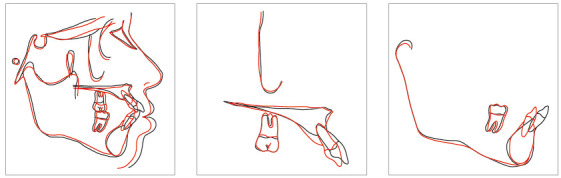
Cephalometric superimposition of Case 4.

The posttreatment facial photographs showed that the reduction of protrusion of incisors contributed positively both to the reduction in chin tension and to improve the lip seal and the smile esthetics, with good exposure of maxillary incisors (Fig. 53). The cephalometric analysis (Tab. 4, Fig. 54) showed a small increase in the FMA angle, reduction in the ANB (from 5.7° to 4.1°), intrusion and retraction of maxillary incisors. The mandibular incisors were intruded and lingually inclined, with improvement in the interincisal angle, which was reduced to normal values. The cephalometric superimposition confirmed that there was bodily retraction and retroclination of the maxillary and mandibular incisors, and also that there was no extrusion of posterior teeth, used as anchorage (Fig. 55).

**Figure 56:**
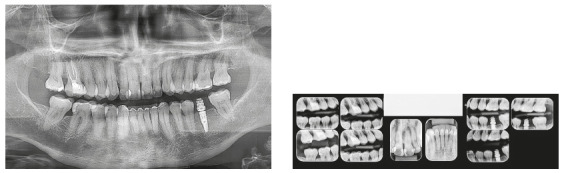
Final radiographs of Case 4.

The treatment results were within acceptable limits, and the patient was satisfied. Periapical and panoramic radiographs (Fig. 56) showed good root parallelism and absence of noticeable root resorption. The radiographs showed a significant reduction in the dimensions of the angular infrabony defects in the maxillary incisors region. During active orthodontic treatment, the probing depths and bone levels in the anterior segment, radiographically assessed, were maintained at the levels reached after initial periodontal treatment.

During the retention control of this patient, there was a great need for speech therapy, due to the atypical tongue posture during rest and speech. It is important to note that, in patients with loss of periodontal support, speech-language disorders can be critical to the stability of treatment and should always be evaluated. The joint performance of Speech Therapy and Periodontics provides these patients with the benefit of interaction between shape and function, since the maladjustments of the static and dynamic structures generate compensations in the performance of oral functions,^47^ which should then be normalized after orthodontic correction. Orthodontic treatment will reestablish a stable position of bones and teeth, to achieve the balance of these structures, while speech therapy directly meets the functional demands resulting from this structural imbalance, interfering with the dynamic structures, thus restoring the functions of the stomatognathic system^47^ ^,^ ^48^ and contributing to the stability, since it will adapt the active and passive functions affected by periodontal disease, which should be adjusted to the new positions achieved after orthodontic treatment. In this patient, speech therapy was performed for eight months, until the articulation of phonemes and the tongue posture at rest were readjusted to the new occlusal arrangement, without generating undesirable forces on the dental structures.

## CONCLUSION

Orthodontic treatment in patients with vertical alveolar bone loss and history of periodontal disease should be planned according to the individual characteristics of each patient, such as: insertion level of teeth that need to be moved; patient's oral hygiene; control obtained from periodontal disease; numbers of missing and/or compromised teeth; and types of pathological migrations present.

However, regardless of the particularities of each case, the sequence of six steps described in the Pyramid of Orthodontic-Periodontal Planning should be used to guide the planning and accomplishment of interdisciplinary treatment focused on malocclusions and the needs of patients with a history of periodontitis.

As described in the presented cases, the levels of the pyramid must be staged and individualized for each patient:

1) Achievement of periodontal health: this should always be the first step. However, the duration of this treatment, the need for periodontal surgery or not, and the frequency of periodontal maintenance depend on each case and will be defined by the periodontist. It should be noted that the frequency of periodontal maintenance can be changed during orthodontic treatment, especially during intrusion movements. Ideally, the periodontist should send a report indicating the date of the next follow-up appointment, so that the treatment may be safely conducted, dividing the responsibilities.
2) Anchorage planning: anchorage is critical in the movement of teeth with insertion loss. It should be checked whether the bone levels of posterior teeth allow their use as anchorage units or not. If not, the use of direct or indirect anchorage with TADs may be indicated. If there is severe bone loss in posterior teeth, the tendency to extrusion and anchorage loss will increase.
3) Biomechanical planning: with the loss of alveolar insertion, there is apical displacement of the CRes, which induces an increase in the tendency of uncontrolled inclinations in the accomplishment of orthodontic movements. Therefore, orthodontic forces must be reduced, proportionally to the severity of bone loss.
4) Planning the intrusion movement: this is a very frequent movement in these patients due to pathological migrations. However, it must be planned so that the force falls close to the CRes of the tooth or group of teeth to be moved.
5) Black triangles: they are also frequent in these patients, due to vertical alveolar bone loss. Usually, they become even more apparent after intrusion movements. The possibilities and limitations of correction must be presented and discussed with the patient during planning.
6) Retention: due to bone loss, the stability of results is critical. To maintain long-term results, it is essential to splint the anterior teeth with fixed retainers (sometimes even premolars), identify whether there is a need to install interocclusal plates (in cases of possible parafunctions), speech therapy treatment and follow-up, and maintain routine periodontal and orthodontic consultations.

The realignment of teeth associated with the correction of traumatic occlusion facilitates the patient's oral hygiene and increases the chances of maintaining the health of the supporting periodontium, even if reduced. It is widely demonstrated by the literature that patients with periodontitis must be monitored and controlled by the periodontist for life. However, after orthodontic-periodontal treatment, regular control by the orthodontist also becomes essential to maintain the occlusion of this patient, since changes in the periodontal health condition may occur, with possible additional bone and dental losses. Therefore, the orthodontist will contribute to the periodontist to maintain the patients' periodontal and occlusal balance, despite the countless potential changes that may occur.
